# Interactions Between Neutrophils and Periodontal Pathogens in Late-Onset Periodontitis

**DOI:** 10.3389/fcimb.2021.627328

**Published:** 2021-03-12

**Authors:** Qingsong Jiang, Yuxi Zhao, Yusen Shui, Xuedong Zhou, Lei Cheng, Biao Ren, Zhu Chen, Mingyun Li

**Affiliations:** ^1^ State Key Laboratory of Oral Diseases, West China Hospital of Stomatology, National Clinical Research Center for Oral Diseases, Sichuan University, Chengdu, China; ^2^ Department of Conservative Dentistry and Endodontics, Guiyang Hospital of Stomatology, Guiyang, China

**Keywords:** polymorphonuclear, neutrophils (PMNs), periodontitis, killing, evasion, *Porphyromonas gingivalis*

## Abstract

Late-onset periodontitis is associated with a series of inflammatory reactions induced by periodontal pathogens, such as *Porphyromonas gingivalis*, a keystone pathogen involved in periodontitis. Neutrophils are the most abundant leukocytes in the periodontal pocket/gingival crevice and inflamed periodontal tissues. They form a “wall” between the dental plaque and the junctional epithelium, preventing microbial invasion. The balance between neutrophils and the microbial community is essential to periodontal homeostasis. Excessive activation of neutrophils in response to periodontal pathogens can induce tissue damage and lead to periodontitis persistence. Therefore, illuminating the interactions between neutrophils and periodontal pathogens is critical for progress in the field of periodontitis. The present review aimed to summarize the interactions between neutrophils and periodontal pathogens in late-onset periodontitis, including neutrophil recruitment, neutrophil mechanisms to clear the pathogens, and pathogen strategies to evade neutrophil-mediated elimination of bacteria. The recruitment is a multi-step process, including tethering and rolling, adhesion, crawling, and transmigration. Neutrophils clear the pathogens mainly by phagocytosis, respiratory burst responses, degranulation, and neutrophil extracellular trap (NET) formation. The mechanisms that pathogens activate to evade neutrophil-mediated killing include impairing neutrophil recruitment, preventing phagocytosis, uncoupling killing from inflammation, and resistance to ROS, degranulation products, and NETs.

## Introduction

In recent decades, governments and nongovernmental associations have made great efforts to improve people’s oral health status. However, the prevalence of severe periodontitis worldwide in 2015 (7.4%) was not lower than that in 1990 (7.4%) (DALYs et al., 2015; Kassebaum et al., 2017). Over the past 100 years, many hypotheses and concepts have been proposed for the microbial etiology of periodontitis. The ‘red complex,’ consisting of *T*a*nnerella forsythia* (*T. forsythia*)*, Treponema d*enticola (*T. denticola*), and *Porphyromonas gingivalis* (*P. gingivalis*), has been considered highly related to periodontitis for a long time (Socransky et al., 1987; Holt et al., 1988; Socransky et al., 1998). This hypothesis suggests that periodontitis is caused by the overgrowth of specific bacteria, at least in significant part. However, the ‘red complex’ pathogenic bacterial species, such as *P. gingivalis* and *T. forsythia*, can also be found in healthy gingival tissues (Riep et al., 2009). *P. gingivalis* cannot strongly promote periodontitis independently (Moore et al., 1982). Therefore, the “red complex” has some limitations in explaining periodontitis mechanisms. Recently, Hajishengallis et al. proposed a new model, polymicrobial synergy and dysbiosis (PSD), to explain the etiology of periodontits (Hajishengallis and Lamont, 2012). In this model, periodontitis is not triggered by a specific single pathogenic species but by some “keystone pathogens” accompanied by the entire microbial community. Keystone pathogens can cause community dysbiosis and elevate community virulence, impairing the immune surveillance and inducing an inflammatory response. Other members within the community play distinct roles that converge to form and stabilize the periodontitis-provoking microbiota. Inflammation and dysbiosis promote each other, leading to the progression of periodontitis (Hajishengallis and Lamont, 2012; Lamont and Hajishengallis, 2015; Shaikh et al., 2018).

As mentioned above, a major causative factor for periodontitis is the colonization of the oral biofilm by opportunistic bacteria. *P. gingivalis* is a major keystone pathogen of periodontitis that manipulates the immune response and causes microbial dysbiosis (Hajishengallis, 2015; Hajishengallis, 2020). Notably, oral bacteria are required but not sufficient to induce periodontitis. It is the result of an exaggerated inflammatory response to the bacterial challenge that leads to periodontal damage (Hajishengallis, 2014a).

Neutrophils, also known as polymorphonuclear leukocytes (PMNs), are the most abundant leukocytes in periodontal pockets, the gingival crevice, and inflamed periodontal tissues. In healthy periodontal tissues, neutrophils mainly reside in the junctional epithelium and the crevicular fluids. PMNs play an essential role in oral health, as they maintain the symbiosis of the bacterial community in healthy periodontal tissues and exert bactericidal effects in periodontitis (Uriarte et al., 2016). Fine et al. found two subsets of oral neutrophils: parainflammatory neutrophils and proinflammatory neutrophils (Fine et al., 2016). They reported that parainflammatory neutrophils are the main subset in a healthy oral cavity, and proinflammatory neutrophils are the main subset in the oral cavity of late-onset periodontitis patients. Compared with parainflammatory neutrophils, proinflammatory neutrophils exhibit elevated phagocytosis, degranulation, ROS production, and NET formation. Neutrophils from periodontal patients release more oxygen radicals than those from periodontally healthy individuals, and the increased reactivity of neutrophils in patients is attributed to the activation of Fcγ-receptor pathway (Fredriksson et al., 2003; Gustafsson et al., 2006; Matthews et al., 2006). Neutrophils in periodontitis also have a higher cytokine reactivity as the expression of chemokines and the release of cytokines (IL-1β, IL-6, IL-8, and TNF-α) are higher in neutrophils isolated from late-onset periodontitis patients (Lakschevitz et al., 2013; Ling et al., 2015). However, during periodontitis, the immune response is a double-edged sword because the cytokines and antimicrobial components produced during the immune response, such as IL-17, IL-6, IL-1β, TNF-α, ROS, and NETs, can induce bone resorption and soft tissue injury (Ramadan et al., 2020; Magan-Fernandez et al., 2020). In periodontitis, neutrophils are recruited through tethering and rolling, adhesion, crawling, and transmigration. Next, neutrophils exert antimicrobial activities *via* four mechanisms, including phagocytosis, degranulation, cytokine, and NET formation (Uriarte et al., 2016). However, bacteria activate different evasion mechanisms to withstand the antimicrobial killing of neutrophils, mainly by inhibiting the recruitment, preventing phagocytosys, uncoupling killing from inflammation, and resisting degranulation products, ROS, and NETs.

Nowadays, studies on oral diseases have increasingly focused on the intimate relationship between physical and mental health. For example, oral ulcerations are associated with gastrointestinal disorders, nutritional deficiencies, and periodontal diseases are associated with diabetes, prostate cancer, cerebral atherosclerosis, and coronary artery disease (CAD) (Napeñas et al., 2020; Patel et al., 2020; Wei et al., 2020; Winning et al., 2020). Late-onset periodontitis is one of the two most prevalent oral diseases (the other is dental caries), and the periodontal condition has been listed as an essential reference of health condition by WHO. This review addresses the mechanisms of neutrophil reaction to periodontal pathogens and the bacterial evasion strategies in late-onset periodontitis.

## Recruitment of Neutrophils

Neutrophil recruitment is considered a multi-step process, including tethering and rolling, adhesion, crawling, and transmigration (Nicu and Loos, 2016) [more details were reviewed by Masgrau et al. (Masgrau-Alsina et al., 2020)].

### Tethering and Rolling

During infection, local vascular endothelial cells are activated by the inflammatory mediators released from the infection site or by pattern recognition receptor (PRR) mediated by the detection of pathogens. The activated endothelial cells upregulate the expression of surface adhesion molecules, initiating the recruitment process (Kolaczkowska and Kubes, 2013). L-selectin is expressed on most leukocytes, including neutrophils, whereas E-selectin and P-selectin are expressed on inflamed endothelial cells (Ley et al., 2007). The binding of E-selectin and P-selectin to their receptor P-selectin glycoprotein ligand 1 (PSGL1) leads to the tethering of neutrophils in the blood to the surface of vascular endothelial cells and their subsequent rolling along the vessel wall (Kolaczkowska and Kubes, 2013).

### Adhesion and Crawling

Rolling along the vessel facilitates neutrophil contact with chemokines deposited on the endothelium. The junctional epithelium surrounding the teeth produces a chemotactic interleukin-8 (IL-8) gradient after contacting oral pathogens (Tonetti et al., 1998; Curtis et al., 2011). Neutrophils express two receptors for IL-8, CXCR1, and CXCR2 (Holmes et al., 1991). IL-8 binding to CXCR1 is associated primarily with IL-8-induced chemotaxis (Wolf et al., 1998), and binding of IL-8 to CXCR2 on neutrophils leads to neutrophil activation, promoting the adhesion to the endothelium. In contrast, tumor necrosis factor can upregulate the expression of endothelial adhesion receptor (Weninger et al., 2000; Hajishengallis, 2020). Once the adhesion is finished, neutrophils crawl along the endothelium to search for an appropriate exit. The crawling of neutrophils is directional and controlled by an intravascular chemokine gradient (Massena et al., 2010).

### Transmigration

Before transmigration, neutrophils are surrounded by endothelial actin-rich microvilli that contain intercellular adhesion molecule 1 (ICAM1) clusters (Barreiro et al., 2002). The endothelial actin structure formation, initiated by RhoA, is necessary to prevent vascular leakage during neutrophil extravasation (Heemskerk et al., 2016). After their exit through the endothelial layer, neutrophils need to pass through pericytes and the vascular basement membrane. This membrane is a continuous layer composed of extracellular matrix (ECM) proteins. Many proteases expressed by neutrophils, such as gelatinase B, elastase, and matrix metalloproteinases, facilitate their exit through the basement membrane (Delclaux et al., 1996; Kuckleburg et al., 2012). Then, neutrophils move towards the pathogens following the chemoattractant gradients, such as IL-8, N-formyl-methionyl-leucyl phenylalanine (fMLP), C5a, and leukotriene B4 (LTB4) (Herrmann and Meyle, 2015).

## Antimicrobial Mechanisms

### Phagocytosis

As the earliest defense against external pathogenetic microorganisms, PMNs respond to bacterial stimuli rapidly. Oral neutrophils in late-onset periodontitis patients have more phagosomes and produce more ROS (Fine et al., 2016). Regulated by cytokines, cellular adhesion molecules (CAMs), and chemokine, PMNs traverse the vascular endothelium after adhesion and chemotactic effect, subsequently arriving at inflammation sites to phagocytose pathogens opsonized by complement and/or IgG and non-opsonized pathogens (Allen and Criss, 2019). The phagocytic action can be significantly enhanced by the opsonization of bacteria with complement or antibodies. Neutrophils engulf bacteria and form intracellular vacuoles known as phagosomes (Rijkschroeff et al., 2018). Then, the intracellular antimicrobial response is activated, which depends on ROS and antimicrobial agents stored in granules.

### Oxidative Burst Response

Oxidative burst response is an effective strategy for infection clearance, accompanied by high oxygen consumption and ROS production through NADPH oxidase complex activation (Nauseef, 2007). The NADPH oxidase is a multicomponent enzyme system consisting of several proteins, such as the flavocytochrome b588 protein, the cytosolic components p40^PHOX^, p47^PHOX^, and p67^PHOX^, and the small GTPase(s) Rac 1 or Rac2 (Belambri et al., 2018). In resting neutrophils, the NADPH oxidase complex is unassembled and inactive. Upon stimulation, these separate components translocate to the membrane to form the enzyme complex with a catalytic activity (Groemping et al., 2003; Nauseef, 2019; Miralda and Uriarte, 2020). The generated ROS can be released into the extracellular space or into the phagosome according to the stimulus. When encountered with a soluble stimulus, the NADPH oxidase complex assembles on the plasma membrane, and the generated ROS is released into the extracellular space. When faced with a particulate stimulus, such as a pathogen, the NADPH oxidase complex assembles on the particle-containing phagosome membrane, and the generated ROS is released into the phagosome (Miralda and Uriarte, 2020). ROS can cause direct oxidative damage to microbes, such as oxidation of methionine residues, lipid peroxidation, base oxidation and deamination, and DNA strand breakage (Paiva and Bozza, 2014).

### Granules

Degranulation has been confirmed in periodontitis, as increased levels of degranulation markers have been detected in periodontitis patients (Nicu et al., 2018). Granules in mature PMNs contain various proteins functioning as antimicrobial host defense, which are histochemically classified as peroxidase-positive and peroxidase-negative. Granules can be functionally distinguished by matrix contents and integral membrane proteins as well (Borregaard and Cowland, 1997). There are four types of granules that can either assemble in bacteria-containing phagosomes or undergo exocytosis to release matrix contents (Borregaard et al., 2007). Secretory vesicles are the easiest to undergo exocytosis, sequentially followed by gelatinase granules, specific granules, and azurophil granules (Nauseef and Borregaard, 2014). Granules contain numerous antimicrobial peptides, such as hCAP-18, lysozyme, metalloproteases (MMPs), myeloperoxidase (MPO), proteinase-3, cathepsin G, and elastase (Borregaard et al., 1983; Klebanoff, 1999; Ujiie et al., 2007; Kobayashi et al., 2018).

### NET Formation

In addition to phagocytosis, respiratory burst response, and granule release, neutrophil extracellular trap (NET) formation is another mechanism developed to defend the host against pathogens. In 2004, Brinkmann et al. described the main composition and structure of NETs and their antimicrobial ability for the first time (Brinkmann et al., 2004). NET is an extracellular fibrillary network produced by activated PMNs, containing DNA, histones, and 30 matrix granule proteins (Brinkmann et al., 2004; Pires et al., 2016). DNA serves as a chelating agent among cations due to its phosphodiester skeleton, possessing intrinsic antimicrobial ability (Mulcahy et al., 2008; Halverson et al., 2015). Histones possess antibacterial ability as well, especially H2A, which is considered as one of the most effective agents (Lee et al., 2009). Additionally, primary granules-derived MPO and neutrophil elastase (NE), secondary granules-derived lactoferrin and PTX3, and tertiary granules-derived MMP-9 and phase pattern recognition receptors (PGRP-S) are also included in the components of NETs (Brinkmann et al., 2004).

As demonstrated by transmission microscopy, scanning electron microscopy, and confocal laser scanning microscopy, there are abundant NETs in the gingival pocket surface and the purulent crevicular exudate of patients with late-onset periodontitis. NETs trap the dispersed subgingival plaque and flow into the oral cavity within the purulent crevicular exudate, preventing the adhesion and invasion to the host gingival tissues (Vitkov et al., 2009; Vitkov et al., 2010). Neutrophils are also attracted toward dental biofilms, where they release NETs to control biofilm formation (Hirschfeld et al., 2015). Additionally, Moonen et al. reported that under the same stimulation, oral neutrophils form 13 times more NETs than neutrophils isolated from peripheral blood (Moonen et al., 2019). However, although NETs play a vital role in the clearance of periodontal pathogens, the harmful effect of excessive formation of NETs on periodontitis should not be ignored. The formation of periodontal pockets interferes with the clearance of pathogen-associated molecular patterns (PAMPs) and damage-associated molecular patterns (DAMPs). These PAMPs and DAMPs trigger excessive NET formation, inducing tissue damage and progression of periodontitis (Vitkov et al., 2017; Vitkov et al., 2020).

## Evasion of Neutrophil-Mediated Destruction by Periodontal Pathogens

### Inhibition of Recruitment

**Figure 1:**
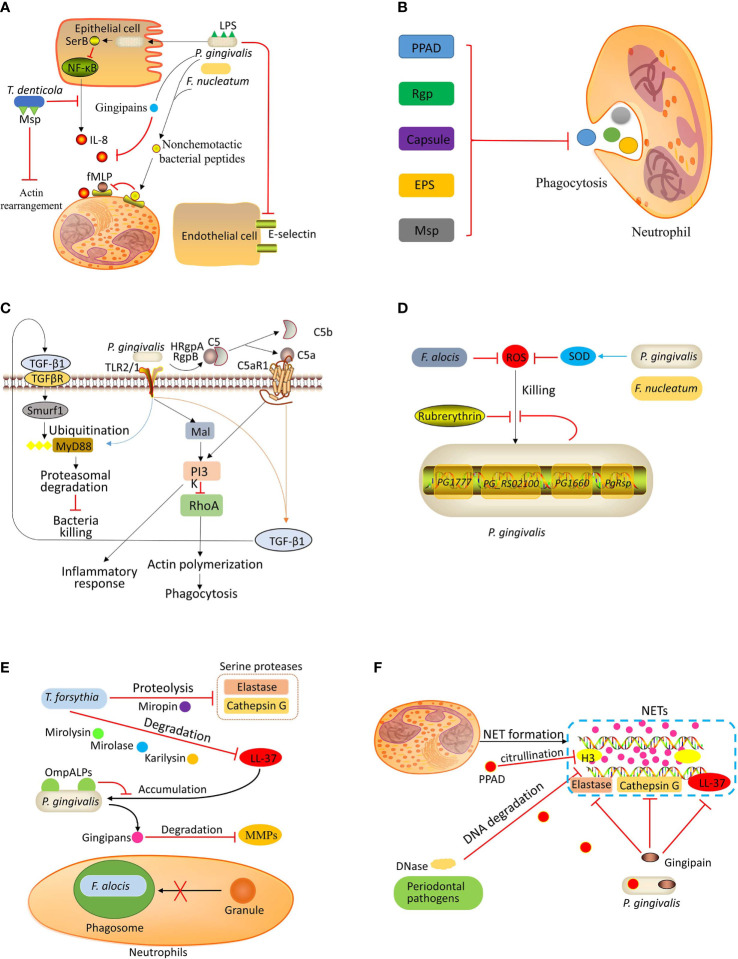
Bacterial evasion strategies. **(A)** Inhibition of recruitment: *P. gingivalis* invades the epithelial cells and secretes SerB, which inhibits the activation of NF-κB, resulting in decreased IL-8 production. The gingipains secreted by *P. gingivalis* can degrade IL-8. *P. gingivalis* and *F*. *nucleatum* can inhibit fMLP-induced chemotaxis by binding nonchemotactic bacterial peptides to fMLP receptors. The LPS of *P. gingivalis* can inhibit E-selectin expression, which is necessary for neutrophil-endothelium interaction. *T. denticola* can suppress the production of IL-8 and degrade IL-8. The major outer sheath protein (Msp) of *T. denticola* can inhibit actin rearrangement, thereby preventing neutrophil recruitment. **(B)** Preventing phagocytosis: *Porphyromonas* peptidylarginine deiminase (PPAD), arginine-specific gingipain (Rgp), capsule, exopolysaccharides (EPS), and Msp contribute to periodontal pathogen evasion from neutrophilic phagocytosis. **(C)** Uncoupling killing from inflammation: *P. gingivalis* coactivates TLR2/1 complex and C5a receptor (C5aR), leading to the release of transforming growth factor‐beta1 (TGF-β1), which induces E3 ubiquitin ligase Smurf1 to ubiquitinate myeloid differentiation primary response protein 88 (MyD88) for subsequent degradation. Moreover, the co-activation of TLR2/1 and C5aR also activates PI3K, which induces an inflammatory response and blocks phagocytosis by inhibiting small GTPase RhoA. **(D)** Resistance to ROS-mediated killing: *P. gingivalis* evades ROS-mediated killing by expressing rubrerythrin and upregulating the expression of the genes *PG1777*, *PG_RS02100*, *PG1660*, and *P. gingivalis* redox-sensing protein (*PgRsp*). *P. gingivalis* and *F*. *nucleatum* can express superoxide dismutase (SOD), which catalyzes the dismutation of superoxides into hydrogen peroxide to produce molecular oxygen. *F*. *alocis* also inhibits ROS production. **(E)** Resistance to granule-mediated killing: *T. forsythia* can degrade LL-37 by karilysin, mirolase, and mirolysin and degrade serine proteases (elastase and cathepsin G) by proteolysis. The outer membrane protein A-like proteins (OmpALPs) of *P. gingivalis* can reduce LL-37 accumulation on the bacterial surface, promoting bacterial resistance to LL-37. The gingipains secreted by *P. gingivalis* can degrade metalloproteases (MMPs). *F. alocis* can inhibit granules recruitment to bacteria-containing phagosome. **(F)** Evasion of killing by NETs: Numerous periodontal bacterial species can secrete DNase to damage the DNA backbone of NETs. In addition, *P. gingivalis* can inactivate antimicrobial components (neutrophil elastase, cathepsin G, and LL-37) of NETs by expressing gingipain. *P. gingivalis* also expresses PPAD to citrullinate histone H3.

Periodontal pathogens inhibit neutrophil recruitment by four main mechanisms: suppressing the production of IL-8, blocking the detection of chemokines, inhibiting the expression of E-selectin, and inhibiting the actin cytoskeleton rearrangement of neutrophils (**Figure 1A**).

Gingival epithelial cells directly contact periodontal pathogens and generate an IL-8 gradient to activate and recruit neutrophils (Hajishengallis and Lamont, 2012). However, *P. gingivalis* can inhibit mRNA accumulation of IL-8 in *F. nucleatum*-stimulated epithelial cells, thereby blocking IL-8 secretion. This effect is dependent on the invasion ability of *P. gingivalis*. Takeuchi et al. (2013) reported that the suppression of IL-8 generation depended on SerB, a serine phosphatase of *P. gingivalis*. The transcription of IL-8 is mainly controlled by NF-κB RelA/p65 homodimer, a transcriptional regulator whose activation depends on phosphorylation (Vallabhapurapu and Karin, 2009). SerB is produced intracellularly to subsequently catalyze the dephosphorylation of NF-κB p65 subunit, thereby diminishing IL-8 production (Takeuchi et al., 2013). Moreover, gingipains secreted by *P. gingivalis* can diminish the activity of IL-8, inhibiting neutrophil chemotaxis (Sochalska and Potempa, 2017). Also, *T. denticola* can suppress the production of IL-8 by primary gingival epithelial cells and degrade IL-8 *via* one of its membrane proteases, dentilisin (Brissette et al., 2008; Jo et al., 2014).

Moreover, the supernatants of *P. gingivalis* and *F. nucleatum* can inhibit fMLP-induced chemotaxis by binding nonchemotactic bacterial peptides to fMLP receptors, making neutrophils unable to detect the chemotactic gradient (Van Dyke et al., 1982; Miralda and Uriarte, 2020). It has also been reported that oral neutrophils express fewer fMLP receptors, and their directional movement toward fMLP is impaired; However, the effect of periodontal pathogens on the downregulation of fMLP receptors expression on neutrophils is unclear (Moonen et al., 2019). These bacteria also inhibit chemotaxis towards endotoxin-activated serum, the active component of which is C5a (Van Dyke et al., 1982; Uriarte et al., 2016). These findings suggest that these pathogens inhibit chemokine detection through at least two independent mechanisms.

Furthermore, LPS isolated from *P. gingivalis* affects the recruitment process. *P. gingivalis*-derived LPS displays a significant amount of lipid A heterogeneity, which contains tetra- and penta-acylated lipid A structures. These two lipid A variants exhibit opposing functions in host response. Penta-acylated lipid A structures promote E-selectin expression while tetra-acylated lipid A structures inhibit E-selectin expression (Darveau et al., 1995; Reife et al., 2006). Since E-selectin mediates the interaction between neutrophils and vascular endothelium (Kolaczkowska and Kubes, 2013), the effects on E-selectin expression could directly influence the recruitment process. Due to opposing effects of tetra- and penta-acylated lipid A on E-selectin expression, *P. gingivalis* can exert a distinct impact on neutrophil recruitment depending on the infection stage. The recruitment of neutrophils is inhibited at the initial stage and stimulated at later stages, contributing to the bacterial colonization at the initial stage and inflammation persistence at later stages (Maekawa et al., 2014; Sochalska and Potempa, 2017).

The major outer membrane sheath protein (Msp), a virulence factor of *T. denticola*, has been reported to inhibit neutrophil chemotaxis (Visser et al., 2013; Jones et al., 2017; Jones et al., 2019). Exposure to Msp inhibits PI3-kinase and activates PTEN (phosphatase and tensin homolog), disturbing the phosphatidylinositol[(3,4,5)]-triphosphate (PIP3) balance (Visser et al., 2013). PI3-kinase and the lipid PTEN play opposing roles in cells to regulate phosphoinositide metabolism. PI3-kinase positively regulates neutrophil chemotaxis while PTEN negatively regulates this process (Subramanian et al., 2007; Heit et al., 2008). During chemotaxis, the accumulation of PIP3 at the leading edge produces polarized negative charges, recruiting Rac1 to the membrane (Magalhaes and Glogauer, 2010). Therefore, exposure to Msp inhibits the location and activation of Rac1 in fMLP-stimulated neutrophils (Magalhaes et al., 2008). Rac1 is the primary GTPase that regulates the actin free barbed end formation, a critical step during actin assembly, in fMLP-stimulated neutrophils (Sun et al., 2007). Thus, Msp of *T. denticola* prevents the actin assembly in neutrophils, which is essential for protrusion formation during transmigration (Thomas et al., 2006). A recent study reported that it is the C-terminal region of Msp that functions as the inhibitor of neutrophil chemotaxis (Jones et al., 2017).

### Prevention of Phagocytosis

Stobernack et al. (2018) reported that *Porphyromonas* peptidylarginine deiminase (PPAD) was important in inhibiting phagocytosis (**Figure 1B**). The internalization level of PPAD-deficient *P. gingivalis* was 2–3 times higher than the W83 strain, and the PPAD-containing culture supernatant and purified recombinant PPAD reduced the internalization of PPAD-deficient *P. gingivalis* by neutrophils. Moreover, an *in vivo* study revealed the role of arginine-specific gingipain (Rgp) in *P. gingivalis*-induced periodontitis in mice (Wilensky et al., 2013). *P. gingivalis* expresses two types of gingipains: Rgp and lysine-specific gingipain (Kgp). Rgp is encoded by two functional genes, *rgpA* and *rgpB*, and Kgp is encoded by a single gene, *kgp* (Nakayama, 1997). Compared with *rgpA*-deficient strain, *rgpA*-expressing *P. gingivalis* induced more alveolar bone loss and less phagocytosis, increasing its survival (Wilensky et al., 2013). The capsule also contributes to bacterial evasion from phagocytosis. *P. gingivalis* capsule inhibits neutrophil phagocytosis not only in mono-species infections but also in mixed infections with *F. nucleatum*. The evasion of capsulated *P. gingivalis* and *F. nucleatum* during mixed infections probably strengthens the virulence since more bone loss was observed in a murine periodontitis model (Polak et al., 2017). Furthermore, the exopolysaccharides (EPS) have also been reported as important in bacterial evasion from phagocytosis (Yamanaka et al., 2011). EPS-producing *P. intermedia* 17 and OD1-16 were rarely engulfed by neutrophils, inducing noticeable lesions in mice. In contrast, EPS-nonproducing bacteria, *P. intermedia* ATCC 25611 and *Porphyromonas gingivalis* strains ATCC 33277, 381, and W83, exhibited a weaker ability in inducing lesions and evasion form phagocytosis (Yamanaka et al., 2011). *T. denticola* was also reported to impair neutrophilic phagocytosis by neutrophils through the action of Msp (Thomas et al., 2006). The mechanism by which Msp inhibits neutrophilic phagocytosis is through the inhibition of actin rearrangement of neutrophils, as detailed in *Inhibition of Recruitment*.

### Uncoupling Killing From Inflammation

Tissue-breakdown products caused by destructive inflammation, such as degraded collagen peptides and heme-containing compounds, can be used as nutrients by periodontal pathogens (Hajishengallis, 2014b). Moreover, treating rabbits with periodontitis using resolvin E1, a pro-resolving mediator of inflammation that suppresses leukocyte recruitment and facilitates resolution, induced inflammation relief and decreased periodontal bacterial load (Hasturk et al., 2007). These findings suggest that immune suppression would not be an optimum strategy for periodontal pathogens to evade immune killing, as less nutrients are available in a non-inflammatory environment. *P. gingivalis* is a low-abundance periodontal pathogen that can alter the number and composition of commensal microbiota. A commensal microbiota was required in *P. gingivalis*-induced periodontitis, as it failed to induce inflammatory bone loss in germ-free mice (Hajishengallis et al., 2011). Maekawa et al. (2014) reported that *P. gingivalis* uncouples neutrophil-mediated bacterial clearance from neutrophil-induced inflammation by manipulating complement and TLR signaling pathways, by which the periodontal bacteria can escape from killing and survive the inflammation (**Figure 1C**). Activation of C5a receptor-1 is a critical step in this pathway. In C5a receptor‐1‐deficient mice or in wild type (WT) mice injected with C5a receptor‐1 antagonist, the survival of *P. gingivalis* and its commensal microbiota and inflammation were suppressed (Hajishengallis et al., 2011; Maekawa et al., 2014). Similarly, *P. gingivalis* loses its ability to induce inflammation and evade killing in TLR-2-deficient mice (Burns et al., 2006). For neutrophils that phagocytose bacteria, the TLR-2 signaling pathway activates PI3K to inhibit phagolysosomal maturation, promoting intracellular survival of *P. gingivalis* (Makkawi et al., 2017). The co-activation of TLR-2 and C5aR depends on *P. gingivalis*-expressed TLR-1/TLR-2 complex ligands and arginine‐specific gingipains, which can cleave C5 to generate C5a (Popadiak et al., 2007). Then, neutrophils release transforming growth factor (TGF-β1), which mediates myeloid differentiation primary response protein 88 (MyD88) ubiquitination and subsequent proteasomal degradation *via* the E3 ubiquitin ligase Smurf1 (Hajishengallis, 2020). Neutrophil-mediated clearance of bacteria in response to *P. gingivalis* infection depends on MyD88, as *P. gingivalis* loads in MyD88^-/-^ mice are significantly higher than that in WT mice (Burns et al., 2010). Therefore, proteasomal degradation of MyD88 is crucial for the evasion of periodontal pathogens. Moreover, the C5a receptor‐1/TLR‐2 crosstalk activates the Mal-PI3K pathway, inhibiting neutrophilic phagocytosis by suppressing actin polymerization mediated by small GTPase RhoA. Meanwhile, the activation of the Mal-PI3K pathway leads to the release of proinflammatory cytokines (e.g., IL-1β, IL-6, and TNF-α) that induce bone resorption in late-onset periodontitis (Maekawa et al., 2014). When the C5a receptor‐1/TLR‐2 crosstalk was inhibited in mice challenged by *P. gingivalis*, the growth of the entire microbial community was prevented (Maekawa et al., 2014). By co-activating C5a receptor‐1 and TLR‐2, *P. gingivalis* activates two pathways to protect the entire dysbiotic community from neutrophil-mediated killing, promoting the persistence of periodontitis.

### Resistance to ROS-Mediated Killing

Neutrophils produce ROS rapidly after exposure to *P. gingivalis* (Jayaprakash et al., 2015). *P. gingivalis* and *F. nucleatum* can catalyze the dismutation of superoxides into hydrogen peroxide to produce molecular oxygen *via* superoxide dismutase (Choi et al., 1991; Diaz et al., 2000; Diaz et al., 2002). Sztukowska et al. (2002) reported that rubrerythrin, an enzyme expressed by *P. gingivalis*, could eliminate intracellular hydrogen peroxide by catalytic reduction, promoting *P. gingivalis* resistance to oxidative stress (**Figure 1D**). Furthermore, rubrerythrin allows the proliferation of *P. gingivalis* in mice with a normal oxidative burst response function. However, this effect disappears in NADPH oxidase–null mice (Mydel et al., 2006). These findings imply that *P. gingivalis* can evade ROS-mediated killing and benefit from ROS-mediated tissue damage since tissue breakdown products can be used as nutriments by this pathogen (Hajishengallis, 2014b). Another enzyme expressed by *P. gingivalis*, alkyl hydroperoxide reductase, also contributes to bacterial resistance to ROS by metabolizing H_2_O_2_ (Diaz et al., 2004; Johnson et al., 2004).

A newly identified periodontal pathogen, *Filifactor alocis*, is also reported to prevent intracellular and extracellular ROS production (Edmisson et al., 2018). Neutrophils can internalize live and heat-killed *F. alocis*. The internalization of live *F. alocis* failed to trigger effective ROS production. However, a robust respiratory burst response was observed when neutrophils were challenged by heat-killed *F. alocis*. Additionally, *F. alocis* did not suppress ROS production induced by other bacteria. These findings indicate that *F. alocis-*mediated inhibition of ROS generation is not a global mechanism but a local phagosomal mechanism. As discussed above, prior to generating ROS, NADPH oxidase components are recruited, assembling at phagosome membrane and/or plasma membrane (Miralda and Uriarte, 2020). Therefore, phagosomes containing *F. alocis* might prevent NADPH oxidase recruitment, but the exact mechanism warrants further studies.

A recent study reported that *P. gingivalis* iron-binding protein PG1777 was associated with resistance to oxidative stress (McKenzie et al., 2016). Under prolonged oxidative stress, the expression of a unique gene cluster *grpE-dnaJ-PG1777-PG1778-PG1779* increased in *P. gingivalis*, and the recombinant PG1777 protein exhibited iron-binding properties and protected DNA from degradation. The gene *PG_RS02100* expressed only during logarithmic growth, and the gene *PG1660* in *P. gingivalis* participate in the oxidative stress response (Phillips et al., 2018; Dou et al., 2018). More recently, a novel redox-sensing transcription regulator was identified as *P. gingivalis* redox-sensing protein (PgRsp) (Smiga and Olczak, 2019). When challenged by oxidative stress, PgRsp can regulate gene expression alongside or cooperate with other transcription factors to resist oxidative stress (Smiga and Olczak, 2019). Although several regulatory proteins have been identified in the oxidative stress resistance pathway, the regulatory network is not clear.

### Resistance to Granule-Mediated Killing

Many antimicrobial proteins are stored in neutrophil granules, including serine proteases (such as elastase, cathepsin G, and proteinase 3), antimicrobial peptides (such as LL-37 precursor and a-defensins.), and other antimicrobial components (Allen and Criss, 2019). The LL-37 levels in the gingival crevicular fluid of late-onset periodontitis patients are higher than healthy individuals (Puklo et al., 2008).

Three proteases expressed by *T. forsythia*, karilysin, mirolase, and mirolysin, can inactivate LL-37. Karilysin, a proteolytically active matrix metalloprotease-like enzyme, can reduce the antibacterial activity of LL-37 against *P. gingivalis* by degrading this peptide (Koziel et al., 2010) (**Figure 1E**). Mirolysin, a member of the pappalysin family of metalloproteases, and mirolase, a subtilisin-like serine protease, can degrade LL-37 in a calcium-dependent manner (Ksiazek et al., 2015a; Koneru et al., 2017). Since LL-37 exerts bactericidal and anti-inflammatory activities by binding to LPS, the degradation of this peptide also abolishes its anti-inflammatory effect and promotes the release of TNF-α, contributing to the development of late-onset periodontitis (Koneru et al., 2017). In addition to *T. forsythia, P. gingivalis* has evolved a mechanism to abolish the bactericidal activity of LL-37 by its outer membrane protein A-like proteins, preventing the accumulation of LL-37 on the bacterial surface (Horie et al., 2018). These bacteria can also inhibit the serine protease activity. A serpin, called miropin, from *T. forsythia* can inactivate a broad range of proteases, including neutrophil elastase and cathepsin G, by proteolysis (Ksiazek et al., 2015b; Goulas et al., 2017); however, its actual contribution to bacterial survival is not clear yet. Additionally, elevated levels of MMPs (MMP-8 and MMP-9) were found in salivary samples of periodontitis patients (Ramseier et al., 2009). Gingipains secreted by *P. gingivalis* can degrade MMP-9 (Bondy-Carey et al., 2013). A recent study suggested that *F. alocis* could inhibit specific and azurophil granule recruitment to the bacteria-containing phagosome, promoting bacterial survival (Edmisson et al., 2018).

### Evasion of Killing by NETs

An in vitro study reported that NETs did not inhibit the growth or survival of some periodontal pathogens, including F. nucleatum, Streptococcus intermedius, Streptococcus sanguinis, Actinomyces viscosus, Veillonella parvula, and Capnocytophaga gingivalis (Hirschfeld et al., 2017). Pathogens implement various strategies to escape NETs, including the formation of a polysaccharide capsule, altering the electric charge of the cell surface, biofilm formation, DNase generation, and inhibiting ROS production (Arazna et al., 2013). Numerous periodontal pathogens can express extracellular DNase to degrade the DNA backbone of NETs, such as P. gingivalis, T. forsythia, F. nucleatum, P. intermedia, Parvimonas micra, Streptococcus constellatus, Campylobacter rectus, and Prevotella nigrescens (Palmer et al., 2012; Doke et al., 2017) (**Figure 1F**). Doke et al. (2017) reported that the DNA degradation activity of P. intermedia was higher than that of P. gingivalis and F. nucleatum. They also revealed that nucleases secreted by P. intermedia were encoded by nucA and nucD, and both Mg^2+^ and Ca^2+^ were required for optimal nuclease activity. F. alocis is also capable of reducing NETs. However, it only reduces NETs formation induced by phorbol 12-myristate 13-acetate (PMA), but not pre-formed NETs (Armstrong et al., 2018). This finding suggests that extracellular DNase expression is not the mechanism of F. alocis to reduce NETs; however, the exact mechanism has not been explained.

Apart from the DNA backbone, NETs contain various bactericidal components, including LL-37, neutrophil elastase, and cathepsin G (Delgado-Rizo et al., 2017). The gingipains of *P. gingivalis* play a dual role in the NET formation, as they mediate NETs formation in response to *P. gingivalis* infection and protect *P. gingivalis* from being entrapped and subsequently destroyed. The serine protease activity of neutrophil elastase and cathepsin G is suppressed in gingipain-induced NETs, and the activity can be partially rescued by gingipain inhibitor, Kyt-1. Similarly, LL-37 and its precursor protein cannot be detected in NETs triggered by gingipain, and this effect can be inhibited by Kyt-1 (Bryzek et al., 2019). These findings suggest that gingipains can inactivate or degrade bactericidal components to reduce the antibacterial activity of NETs.

Furthermore, *P. gingivalis* secretes PPAD to citrullinate the histone H3 and inhibit the antimicrobial activity of NETs (Stobernack et al., 2018).

## Conclusion

Approximately 7.4% of the world population suffer from severe periodontitis. Periodontitis is associated with several factors, such as smoking, diabetes, age, and immune deficiency. Current studies support that periodontitis is initiated by periodontal bacteria, and neutrophils are the primary immune cells recruited to exert an antibacterial effect. Several antimicrobial patterns of neutrophils have been identified, including respiratory burst response, phagocytosis, degranulation, and NET formation. However, periodontal bacteria have evolved strategies to evade neutrophil-mediated killing, which lead to the persistence of inflammation. As inflammation continues, the inflammatory factors and antibacterial components can induce severe tissue damage. Therefore, preventing bacterial evasion might be a potential preventive action in late-onset periodontitis. As periodontal pathogen evasion depends on some key components, such as LPS, nonchemotactic bacterial peptides, HRgpA, RgpB, rubrerythrin, miropin, karilysin, mirolase, mirolysin, OmpALPs, PPAD, and DNase, inhibition the production, degradation, and irreversible binding of these specific components might be potential therapeutic targets to prevent bacterial evasion.

## Author Contributions

QJ, YZ, and YS drafted the manuscript. XZ, LC, BR, ZC and ML edited and added the valuable insights into the manuscript. All authors contributed to the article and approved the submitted version.

## Funding

This study was supported by the National Key Research and Development Program of China 2016YFC1102700 (XZ), the Sichuan Science and Technology Program 2021YFH0188 (ML), the National Natural Science Foundation of China grant 81400501 (ML), 81870759 (LC), and 81430011 (XZ), Guiyang Science Plan Project, Guizhou Province of China [2018]1-59 (ZC), the Youth Grant of the Science and Technology Department of Sichuan Province, China 2017JQ0028 (LC), and the Innovative Research Team Program of Sichuan Province (LC).

## Conflict of Interest

The authors declare that the research was conducted in the absence of any commercial or financial relationships that could be construed as a potential conflict of interest.
